# Endemic Fungal Infective Endocarditis Caused by Coccidioides, Blastomyces and Histoplasma Species in the United States

**DOI:** 10.7759/cureus.60285

**Published:** 2024-05-14

**Authors:** Sabirah N Kasule, Leah M Grant, Michael A Apolinario, Lisa J Speiser, Christopher F Saling, Janis E Blair, Holenarasipur R Vikram

**Affiliations:** 1 Infectious Diseases, Mayo Clinic, Phoenix, USA; 2 Cardiology, Mayo Clinic, Phoenix, USA

**Keywords:** endemic infections, valvular heart disease, fungal infection, histoplasma, histoplasmosis, endocarditis, coccidioidomycosis, coccidioides, blastomyces, blastomycosis

## Abstract

We describe a recent case of *Coccidioides* bioprosthetic aortic valve infective endocarditis successfully managed at our institution. This led us to perform a literature review of endemic fungal infective endocarditis in the United States caused by *Coccidioides*, *Blastomyces*, and *Histoplasma*. Symptoms preceded infective endocarditis diagnosis by several months. Patients with *Coccidioides* and *Blastomyces *infective endocarditis were younger with fewer comorbid conditions. Valvular involvement was relatively uncommon in *Blastomyces *infective endocarditis (27%). Fungemia was noted in patients with infective endocarditis due to *Histoplasma *(30%) and *Coccidioides *(18%). Mortality rates for infective endocarditis were high (*Histoplasma*, 46%; *Coccidioides*, 58%; *Blastomyces*, 80%); infective endocarditis was commonly diagnosed post-mortem (*Coccidioides, *58%; *Blastomyces, *89%). Most surviving patients with infective endocarditis (*Histoplasma,* 79%; *Coccidioides,* 80%) underwent valve surgery along with prolonged antifungal therapy. The two surviving patients with *Blastomyces *infective endocarditis received antifungal therapy without surgery.

## Introduction

Infective endocarditis (IE) is an uncommon manifestation of endemic fungal infections in the United States. We describe the clinical presentation, challenges with diagnosis, and management of a patient with prosthetic valve *Coccidioides *IE (CIE). We conducted a literature review of all cases of CIE and *Blastomyces *IE (BIE). The *Histoplasma *IE (HIE) review was recently published and will be briefly summarized.

## Case presentation

A 61-year-old man with a prior history of mechanical aortic valve (AV) replacement for aortic stenosis (2008), atrial fibrillation, and chronic kidney disease sought care at an external facility for cough, dyspnea, and fevers in June 2019. Computed tomography (CT) of the chest showed bilateral nodular and ground-glass opacities and mediastinal lymphadenopathy. After discharge, his *Coccidioides *complement fixation (CF) titer was reported as 1:32 (ref: negative). *Coccidioides *immunoglobulin M by enzyme immunoassay and immunoglobulin G by immunodiffusion were also positive.

He returned to that hospital in July 2019 with lightheadedness, dizziness, fevers, and nausea. Chest CT showed improved pulmonary nodularity. Head CT was unremarkable, and the CF titer was 1:32. He was discharged with a treatment regimen of fluconazole 200 mg/d (given underlying chronic kidney disease) until August 2020.

In November 2020, he sought care at our institution for syncope, and new peripheral eosinophilia was identified. Chest CT showed a few subcentimeter opacities; head CT was unremarkable. The *Coccidioides *CF titer was elevated at 1:256. Fluconazole 400 mg/d was initiated given significant escalation in CF titer following its discontinuation in August 2020, indicating relapsed infection.

He was readmitted 10 days after discharge for ataxia and falls. He had a new left-sided facial droop, left-sided dysmetria, and bilateral intention tremor. Magnetic resonance imaging (MRI) of the brain showed small, bilateral, subacute cerebellar infarcts. Transthoracic echocardiography (TTE) showed an ill-defined mobile echodensity on the AV. Transesophageal echocardiography (TEE) done five days later was suboptimal due to acoustic shadowing. Immobile density at the sinotubular junction was felt to reflect calcification. Due to valve shadowing, vegetation could not be excluded. Cerebrospinal fluid (CSF) analysis showed 10.6 nucleated cells/mL (83% lymphocytes, 0% neutrophils, 0% eosinophils) with normal protein and glucose values and negative findings of CSF *Coccidioides *serologic analysis and polymerase chain reaction. The results of blood cultures were negative. The serum CF titer remained at 1:256 (ref: negative). The patient’s neurologic symptoms resolved within three days. Given a finding of QTc prolongation, fluconazole was switched to isavuconazole 372 mg/d. His embolic stroke was attributed to inconsistent anticoagulation and atrial fibrillation.

During follow-up evaluation, a bone scan was negative and CF titers had decreased to 1:128. Because of unexplained CSF pleocytosis and central nervous system (CNS) symptoms, the isavuconazole dose was increased.

In February 2021, the patient was again hospitalized at our institution with fevers, altered mental status, and new leukocytosis to 11.9^10(9)/L (ref: 3.4-9.6^10(9) x L). CSF analysis showed 12.8 nucleated cells/mL (ref: 0.0-5.0/microL) (61% lymphocytes, 5% neutrophils, 0% eosinophils). CSF bacterial and fungal cultures, polymerase chain reaction for *Coccidioides*, and fungal metagenomics testing were negative. MRI showed new, subacute embolic infarcts throughout both cerebral hemispheres. Neither TTE nor TEE demonstrated evidence of valvular vegetation or intracardiac thrombus. The blood culture incubation time was extended. Isavuconazole dosage was decreased because of medication intolerance. He was discharged after returning to baseline functional status.

**Figure 1:**
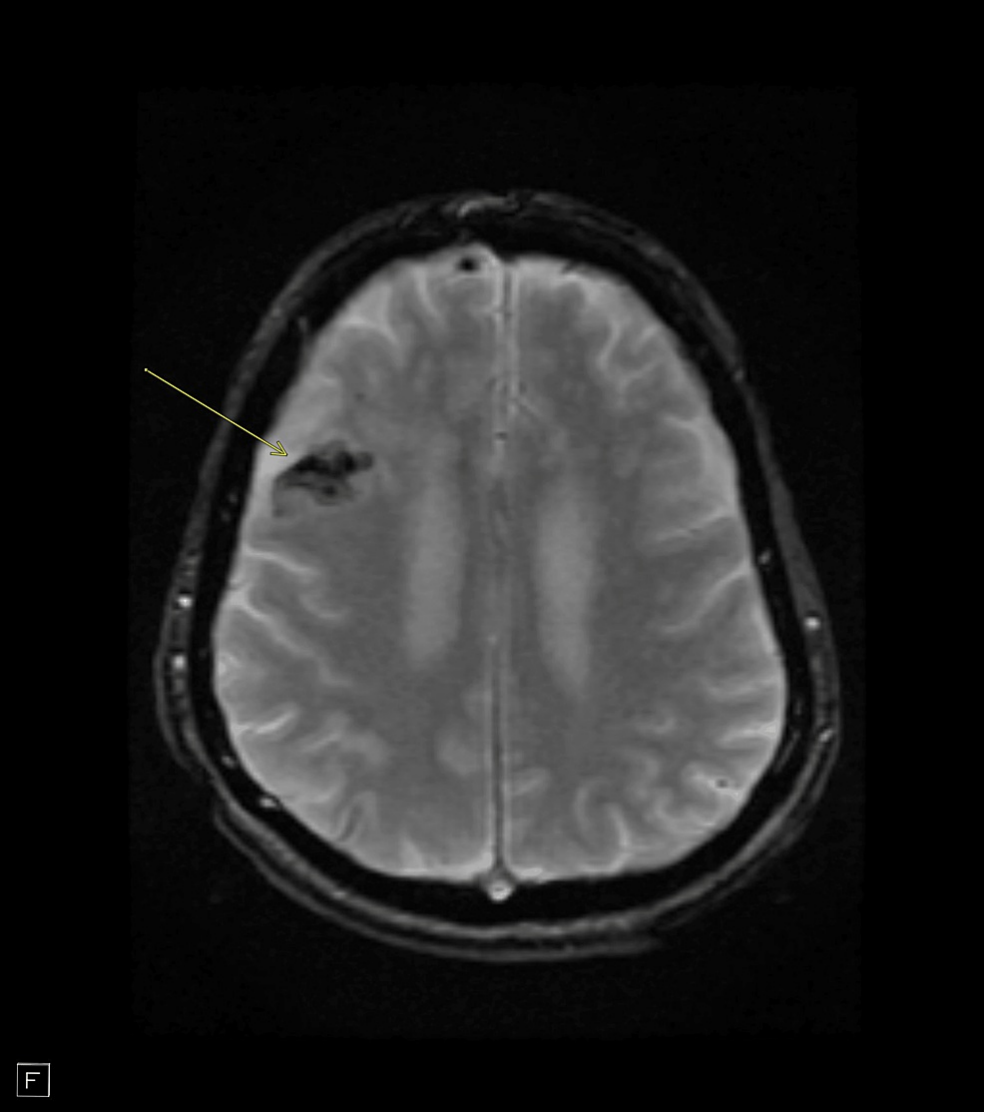
MRI brain without IV contrast showing a new area of right frontal subarachnoid hemorrhage (axial T2 image, arrow). Multiple additional foci of restricted diffusion reflecting embolic infarcts were noted in other cuts.

**Figure 2:**
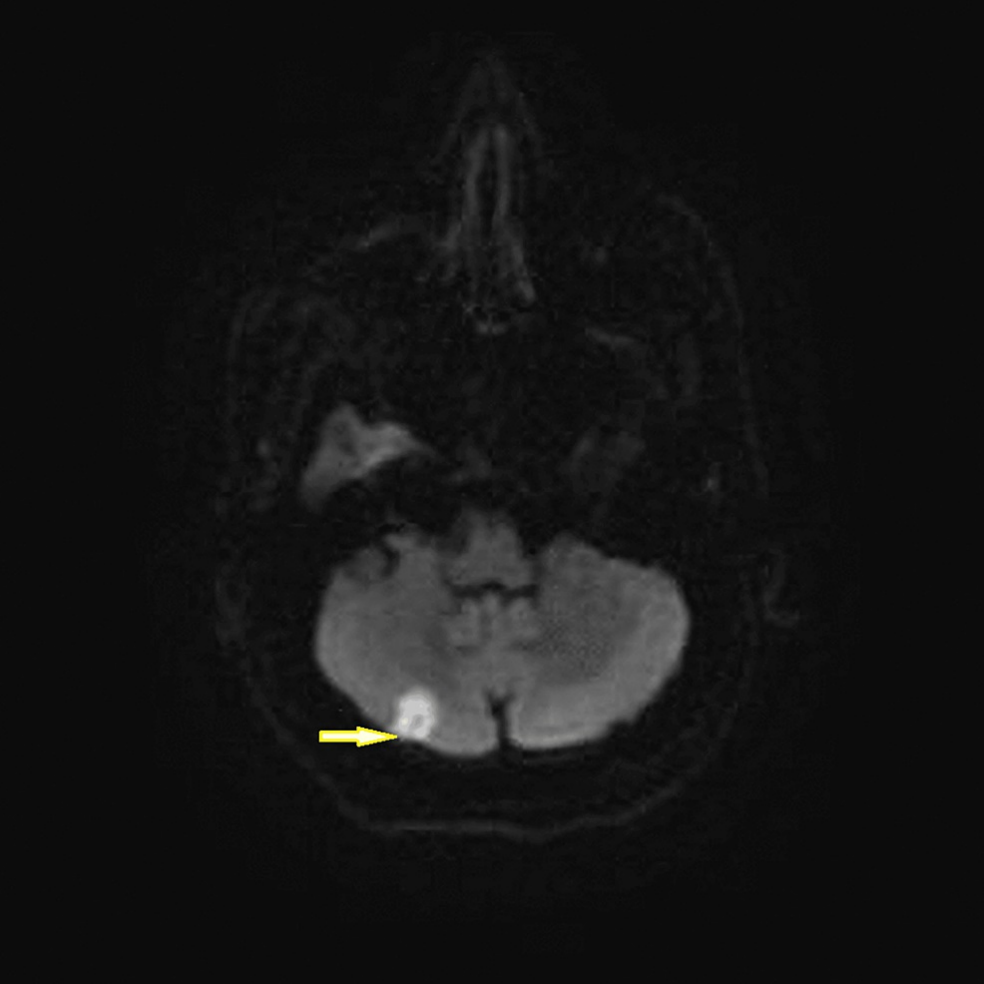
MRI brain without IV contrast with new right cerebellar infarct (axial T2 image, arrow).

Three days later, he was readmitted with weakness and chills. Four sets of blood cultures from his previous admission showed *Coccidioides immitis*/*posadasii*, with a median time to detection of 108.2 hours (range: 106-120 hours). Head CT showed a new, right frontal subarachnoid hemorrhage. Brain MRI showed a new right cerebellar infarct and numerous supratentorial embolic infarcts in the frontal, parietal, right temporal, and left occipital lobes (Figures 1-2). TTE now showed multiple, mobile, nodular echodensities attached to the aortic prosthetic sewing ring. TEE confirmed these findings, with the largest vegetation measuring 1.0×0.6 cm.

**Figure 3:**
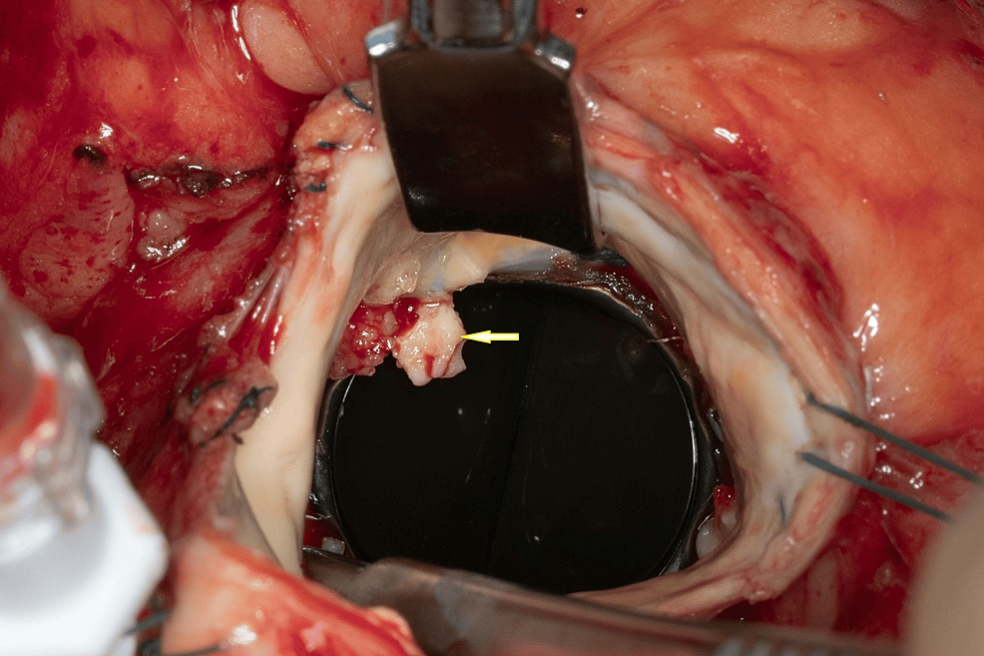
Intra-operative view of infected metallic aortic valve with vegetations along sewing ring (arrow).

He was started on liposomal amphotericin B 10 mg/kg/d and posaconazole 300 mg/d. Combination antifungal therapy is often utilized in the initial phase for severe and disseminated coccidioidomycosis as amphotericin B is fungicidal. Surgical intervention was delayed by a week due to subarachnoid hemorrhage. The patient underwent resection and removal of infected mechanical AV, debridement of inflammatory tissue followed by bioprosthetic AV replacement (Figure 3). No obvious root abscess was noted. Intraoperative tissue cultures grew *Coccidioides *species. Minimum inhibitory concentrations (MIC) for his *Coccidioides *strain were as follows, with no established interpretive guidelines: amphotericin 2 mcg/mL, fluconazole 2.0 mcg/mL, itraconazole 1.0 mcg/mL, posaconazole 1.0 mcg/mL, and voriconazole 0.12 mcg/mL. His HIV testing was negative.

After surgery, he completed eight weeks of posaconazole and liposomal amphotericin B combination therapy. Eight months later, his CF titers had decreased to 1:4 and were negative as of March 2023. Posaconazole was switched to isavuconazole in February 2023 because of resistant hypertension from posaconazole-related mineralocorticoid excess. He continues to do well without evidence of endocarditis or its sequelae and is planned for lifelong isavuconazole therapy.

Methods

We queried PubMed, Scopus, Google Scholar, and Ovid MEDLINE for publications from 1900 through 2022. Keywords included “*Coccidioides*,” “coccidioidomycosis,” “*Blastomyces*,” “blastomycosis,” “endocarditis,” “cardiac mass,” “thrombus,” “cardiac involvement,” “disseminated,” and “valley fever.” Only cases with definite IE or cardiac involvement based on the author’s report and/or modified Duke Criteria for IE were reviewed.

A comprehensive review of HIE was recently published by Boyanton et al. [1]. We referred to this publication for specifics pertaining to HIE.

The earliest reference to CIE was made by Evans and Ball [2] in 1929 who noted “endocarditis due to [*Coccidioides*] *immitis*” in a case series of 50 *Coccidioidal granulomas*. In addition to our current case, 12 other cases of CIE have been published in the literature. We included only 11 cases for which adequate descriptive data were available [3-12].

Cardiac involvement with *Blastomyces *sp. appears to be extremely rare. Our literature review identified 19 potential cases of BIE, but seven of these cases [13-16] described dissemination to the heart without further specifics. Two references described the same case [17,18]. Our final cohort consisted of 11 cases with definite endocardial involvement [17-26].

Results

Coccidioides Infective Endocarditis (CIE)

Of 12 identified cases of CIE, eight patients were male, five were White, and eight resided in California (Table 1). The rest lived in Arizona or Utah. In the case of donor-derived CIE, the donor was from Nevada [11]. Five patients were diagnosed before 1980, all post-mortem. The other seven patients were diagnosed after 2000. The median age at diagnosis was 34 years (range: 3 weeks to 61 years). Two of the 11 patients with information available were immunocompromised (heart transplant and juvenile inflammatory arthritis). One patient was postpartum. The heart transplant patient was hospitalized for CIE 10 weeks post-transplant [11]. The patient with juvenile inflammatory arthritis received methotrexate 10 months before CIE [8]. The postpartum patient had primary pulmonary disease followed by disseminated coccidioidomycosis with previous pregnancies and was intermittently compliant with azole therapy. CIE developed two months after a spontaneous abortion [3].

**Table 1:** Coccidioides infective endocarditis case descriptions AMB: amphotericin B; AR: aortic regurgitation; AS: aortic stenosis; AV: aortic valve; AVR: aortic valve replacement; B: Black; BP: bioprosthetic; CF: complement fixation; CKD: chronic kidney disease; DM II: type 2 diabetes; F: female; FLU: fluconazole; H: Hispanic; IE: infective endocarditis; ISAVU: isavuconazole; ITRA: itraconazole; LA: left atrium; M: male; MV: mitral valve; N: no; Neg: negative; NR: not reported; path: pathology; PMH: past medical history; POSA: posaconazole; PV: pulmonic valve; RA: right atrium; SVC: superior vena cava; TTD: time to detection; TV: tricuspid valve; veg: vegetation; W: White; Y: yes

Reference	Age, y/sex/ race	State of residence	PMH	TTD	IE finding	Positive test results		CF titer	Treatment	Outcome
						Blood cultures	Endocardial path			
Abbott and Cutler, 1936 [12]	34/M/B	CA	None	60 h	“Coccidioides endocarditis”	NR	NR	NR	None	Died
Epstein, 1938 [5]	M	CA	NR	NR	Multiple veg on “valves”	Y	Y	NR	None	Died
Townsend and McKey, 1953 [10]	0.06/F/W	CA	None	2 mo	Veg on MV and TV	N	N	1:1 (4+)	None	Died
Merchant et al., 1958 [9]	21/M/B	CA	Coccidioides skin abscesses	10 mo	Abscess at MV	N	Y	NR	None	Died
Olavarria and Fajardo, 1971 [6]	37/M/W	CA	None	4 mo	Veg on MV	NR	Y	1:256	AMB	Died
Reuss et al., 2004 [7]	53/M/H	UT	AS after BP AVR, pulmonary Coccidioides	10 wk	Veg on BP AV	N	Y	1:32	AMB 1.2 g, FLU 800 mg/d lifelong, AVR	Survived
Reuss et al., 2004 [7]	40/M/W	AZ	DM II, CKD, pulmonary Coccidioides	3 y	Veg on MV, AV, TV, PV	N	Y	1:2	None	Died
La Via et al., 2005 [8]	10/M/H	CA	Juvenile inflammatory arthritis	6 mo	2×1.4-cm pedunculated mass originating from RA wall-SVC junction protruding through TV into RV; no actual involvement of TV	NR	Y	1:2,048	AMB 5 mg/kg/d for 3 mo, then 5 mg/kg/d 3´/wk for 3 mo, then ITRA 300 mg twice daily lifelong, thrombectomy	Survived
Aronowitz, 2014 [4]	19/F/B	CA	None	2 y	Mass from SVC into RA	NR	Y	NR	AMB, FLU	Survived
Horng et al., 2015 [3]	34/F/H	CA	Pulmonary Coccidioides (’04) with dissemination lungs/liver (’07) during pregnancy	2 mo	New AR, mediastinal mass, AV-positive histology/culture	N	Y	1:8	AVR, POSA lifelong	Survived
Nelson et al., 2016 [11]	52/F/W	Donor, NV Recipient, CA	Heart transplant	4 wk	Mycotic aortic aneurysm at anastomotic site with involvement of pericardium, RV myocardium, pulmonary artery trunk, LA anastomosis, RA thrombus	N	Y	Neg	None	Died
Current report	61/M/W	AZ	Pulmonary Coccidioides, AVR, CKD	3 mo	Multiple veg on the aortic prosthetic sewing ring	Y	Y	1:256	AMB 10 mg/kg/d, then 5 mg/kg/d for 8 wk, POSA 300 mg/d with switch to lifelong ISAVU, AVR	Survived

Five patients had a previous diagnosis of coccidioidomycosis; four with pulmonary infections and one with skin abscesses. Three of four with pulmonary coccidioidomycosis were in remission before CIE diagnosis. The median duration between coccidioidomycosis diagnosis and CIE was 10 months (range: 6-108 months). Except for the patient with disseminated disease during her pregnancies [3] and the other with uncontrolled disease, no patients with prior coccidioidomycosis had initiation, alteration, or escalation of immunosuppression at the time of CIE.

The median time to CIE diagnosis after symptom onset was three months (range: 10 weeks to 3 years). Two of 11 cases (18%) had positive blood cultures. Eleven of the 12 cases had confirmatory cardiac histopathologic findings. Endocardial cultures were positive for *Coccidioides *in five of six cases. Seven cases were diagnosed post-mortem; four of these had no prior cardiac imaging owing to lack of availability or lack of initial suspicion [5,6,10,12], including one reported by Olavarria and Fajardo [6] with known disseminated coccidioidomycosis and another by Epstein [5] that had positive blood cultures for *Coccidioides *species. Among the other three cases, two had endocardial lesions on TTE, but blood cultures were repeatedly negative [7,11], and one case had two negative TTEs and 10 sets of negative blood cultures despite ongoing embolic phenomena to his hands and feet [9].

Four of the five cases diagnosed with antemortem had evidence of endocarditis on initial TTE or TEE. In the final case, our patient had nonspecific findings on initial TTEs and TEEs before vegetations were visualized. In this antemortem cohort, only our case had positive *Coccidioides *blood cultures; the rest were diagnosed by histologic analysis or cardiac tissue cultures.

CF titers were available for all but four cases (range: negative to 1:2,048). No obvious correlation was noted between CF titer, diagnosis of CIE, and survival. Of eight cases with valvular IE, the mitral (n=4) and aortic (n=4) valves were most often affected; three cases had multivalvular involvement. Two cases involved prosthetic AVs. Three cases demonstrated mural endocarditis. One case had endocarditis of unspecified location on autopsy [12].

Eleven of 12 cases of CIE had evidence of extracardiac manifestations (Table 1), with the CNS representing the most common site. One case by Reuss et al. [7] may have had CNS involvement but was omitted from the final tally given the lack of details. Ours was the only case of confirmed CNS embolic phenomena. Ten of 12 cases reported pulmonary involvement, which most likely represented the primary site of infection.

Seven of the 12 cases of CIE were fatal. None of the patients who died underwent surgical intervention. Only one received amphotericin because four were diagnosed before its availability in 1960, and five were diagnosed before fluconazole availability in 1988. Two patients had concurrent contributors to death including streptococcal meningitis [12] and myocardial infarction [7]. Of the surviving patients, four of the five had surgery (valve replacement or endocardial lesion resection). Four received amphotericin B and azole combination therapy, and one was treated with azole monotherapy. For the four patients with reported outcomes, lifelong azole suppression was used.

Blastomyces Infective Endocarditis (BIE)

Eight of 11 patients with BIE (72%) were diagnosed before 1960 (Table 2). Most patients with data available were male (8 of 10) with a median age of 24 years (range: 17-61 years). All patients were diagnosed in or had traveled to an endemic area. One patient had juvenile rheumatoid arthritis and was receiving immunosuppression and another was 22 weeks pregnant. These two were the only female patients and the only two who survived. The other patients were described as previously healthy.

**Table 2:** Blastomyces infective endocarditis case descriptions AMA: against medical advice; B: Black; BAL: bronchoalveolar lavage; F: female; ITRA: itraconazole; LA: left atrium; LAA: left atrial appendage; LV: left ventricle; M: male; NR: not reported; PMH: past medical history; PTA: prior to admission; RA: right atrium; RAA: right atrial appendage; STI: sexually transmitted infection; SVC: superior vena cava; TTD: time to detection; W: White

Reference	Age, y/sex/race	State of residence	PMH	TTD	Endocardial involvement	Positive tests	Treatment	Outcome
Bechtel and LeCount, 1914 [19]	38/M/W	IL	Previously healthy. 4 mo PTA new-onset hemoptysis and multifocal skin abscess + hip abscess later determined to be from Blastomyces	6 mo	Fibrinous mural endocarditis	Histopathology, skin abscess culture	None	Died
Hurley, 1916 [18]; Wade and Bel, 1916 [17]	61/M/W	LA	Previously healthy. Initially seen for 3 mo of cough. Treated for scalp abscess and “bronchial asthma” then left AMA	10 mo	Necrotic LV wall; RAA lesion extending into the endocardium, numerous nodules in the endocardium	Histopathology	None	Died
Coupal, 1924 [20]	24/M/B	VA	Previously healthy. Abscess skin 10 d PTA	4 mo	Multiple LV abscesses, LAA abscess	Histopathology	None	Died
Medlar, 1927 [21]	19/M/W	WI	Previously healthy. Productive cough and nonhealing chest ulcer 3 mo PTA	8 mo	Small tubercle in heart	Histopathology	None	Died
Medlar, 1927 [21]	60/M/W	WI	Muscular rheumatism, otherwise healthy. Admitted 4 mo prior with decompensated heart failure	12 mo	Chronic valvular endocarditis	Histopathology lungs, kidney, spleen	None	Died
Baker and Brian, 1937 [22]	17/M/B	NC	Recurrent Blastomyces chest abscess starting 2 y prior	9 mo	Blastomycotic tubercle in the RA extending into the endocardium	Histopathology	None	Died
Baker and Brian, 1937 [22]	24/M/B	NC	Previously healthy	13 mo	RA lesion pericardium, myocardium	Histopathology	None	Died
Pond and Humphreys, 1952 [23]	33/M/B	TX	Previously healthy, other than 2 episodes of STI	17 d	Mediastinal tumor infiltrating pericardium, destroying LAA, and penetrating to the LA to the endocardial surface. Separate lesion penetrating SVC	Histopathology	None	Died
Light et al., 2008 [24]	NR	Manitoba or Ontario, Canada	NR	NR	Aortic valve	NR	NR	NR
Richards et al., 2018 [25]	20/F/NR	WI	Juvenile rheumatoid arthritis	NR	Intracardiac mass	Histopathology, urine antigen, serum antigen	Antifungal therapy	Survived
Walker et al., 2023 [26]	21/F/B	OH	Previously healthy, 22 weeks pregnant	NR	3-cm mass attached to tricuspid valve	Skin biopsy, BAL	Liposomal amphotericin B, ITRA	Survived

Five patients had subcutaneous abscesses up to two years preceding BIE diagnosis, whereas two had a persistent cough or pneumonia for three months. One patient had hemoptysis and a cutaneous abscess [19]. The median time to BIE diagnosis was 10 months (range: 0.5-13 months). No cases reported positive cultures of blood or valves. Blastomyces antigen testing was inconsistently performed and was positive in only one case [25].

Seven cases described positive cardiac histopathologic findings involving the endocardium. In the other four cases, two were managed medically with diagnosis based on echocardiography, histology, and culture growth from other sites of dissemination, followed by regression of cardiac lesions with azole therapy [25,26]. The third patient had recurrent heart failure. Autopsy findings included “chronic valvular endocarditis” and mitral and tricuspid valvular insufficiency. The authors discussed histopathologic dissemination to lung, kidney, and spleen [22]. The fourth patient had AV endocarditis, but no additional information was provided [24].

Eight of the 11 patients had mural endocarditis without valvular involvement. Manifestations included intracardiac masses or abscesses involving atria, ventricles, or atrial appendages with epicardial, myocardial, and pericardial involvement. Valvular IE was noted in only three cases: one aortic [24], one tricuspid [26], and one unspecified but presumed mitral and/or tricuspid valve given valvular insufficiency [21]. There were no cases of prosthetic valve endocarditis (PVE).

Eight cases of BIE were diagnosed post-mortem; several had positive antemortem *Blastomyces *cultures from cutaneous abscesses or respiratory secretions. Two of the 11 cases had echocardiography with evidence of IE. The skin was the most common site of dissemination (Table 3). Ten patients had evidence of pulmonary involvement.

**Table 3:** Sites of dissemination in CIE and BIE cases BIE: *Blastomyces *infective endocarditis; CIE: *Coccidioides *infective endocarditis; CNS: central nervous system Ten of 12 reported CIE and 10 of 10 reported BIE cases had obvious pulmonary involvement, which was most likely the primary site of infection.

Sites of dissemination	CIE (n=11)	BIE (n=10)
Eye	2	0
CNS	6	3
Chest wall/mediastinum	4	4
Lymph node	2	3
Liver	5	1
Spleen	2	4
Stomach	1	0
Kidney	3	4
Adrenal gland	3	1
Thyroid	2	0
Skin	4	7
Muscle	1	2
Bone	0	6
Genitourinary	2	1
Pancreas	0	1

Eight of the 11 cases were fatal. None of the patients who died received antifungal therapy because the cases predated amphotericin B and azoles, and none underwent valve surgery. The two surviving patients received antifungals; one received liposomal amphotericin B and itraconazole, but the other’s therapy was not disclosed. The disposition of one case is unknown [24].

Histoplasma Infective Endocarditis (HIE)

The 2021 review by Boyanton et al. [1] detailed 61 cases of native and prosthetic valve HIE between 1940 and 2020 (36 native valves and 25 prosthetic valves). The majority (82%) were male and acquired histoplasmosis in endemic regions, except for one case diagnosed in England and one in France. A 2008 review of histoplasmosis in Europe suggests endemic areas on the continent [27]. Only two cases were excluded due to mural endocarditis, one of which is discussed in a 2014 review by Riddell et al. [28].

In the Boyanton et al. [1] review, the median age was 55.5 (range: 17-87) years, and initial symptom onset occurred at a median of 5.5 (range: 0.5-24) months before HIE diagnosis. The AV was most often involved (67%), followed by the mitral valve (32%). Seven of 23 patients (30%) had positive blood cultures. The range of *Histoplasma *antigen positivity was 0% to 50% in the serum and 67% to 90% in the urine. Serologic studies were frequently positive. The overall mortality rate was 46%: 100% without antifungal therapy or surgery (18/18), 50% with antifungals alone (6/12), and 0% with the combination of valve surgery and antifungal therapy (0/22) [1].

Comparison of CIE, BIE, and HIE

*Histoplasma* was the most common cause of endemic fungal IE (Table 4). CIE and BIE affected younger patients with fewer comorbid conditions. HIE seemed to particularly affect prosthetic valves, whereas BIE frequently caused mural endocarditis. Immunosuppression was not a requirement for or a major contributor to cardiac involvement. All patients had nonspecific, indolent symptoms or extracardiac manifestations leading to extended delays in IE diagnosis. Fungemia was common with HIE. Treatments varied substantially among CIE, BIE, and HIE (Table 4). BIE had the highest mortality rate, followed by CIE and HIE.

**Table 4:** Comparison of HIE, CIE, and BIE cases BIE: *Blastomyces *infective endocarditis; CIE: *Coccidioides *infective endocarditis; HIE: *Histoplasma *infective endocarditis; NA: not applicable; NR: not reported; TEE: transesophageal echocardiography; TTE: transthoracic echocardiography ^a^ Values are median (range) or no. of patients/no. available.

	Value^a^		
Characteristics	HIE (n=61)	CIE (n=12)	BIE (n=11)
Demographics			
Age, years [median (range)]	55.5 (17-87)	34 (0.06-61)	24 (17-61)
Sex	(n=60)		(n=10)
Male	49	8	8
Female	11	4	2
Race		(n=11)	(n=9)
White	NR	5	4
Black	NR	3	5
Hispanic	NR	3	0
Immunosuppressed	NR	3	2/10
Time to diagnosis, months [median (range)]	5.5 (0.5-41)	3 (0.08-10)	10 (0.5-13)
Diagnosis		(n=11)	
Mural endocarditis	NA	4	8
Valvular endocarditis	61	8	3
Native valve	36	6	3
Prosthetic valve	25	2	0
Diagnostics			
Positive TTE/TEE	NR	7/8	2/2
Positive blood cultures	7/23	2/11	0
Positive endocardial cultures	13/27	5/6	0
Positive endocardial histopathology	NR	11/12	10/11
Positive histopathology/culture (other)	19/40	5/7	6/10
Treatment	(n=52)		(n=10)
None	18	6	8
Antifungals only	12	2	2
Antifungals + surgery	22	4	0
Outcomes			
Overall mortality	24/52	7/12	8/10
Mortality, no treatment	18/18	6/6	8/8
Mortality, antifungals only	6/12	1/2	2/2
Mortality, antifungals + surgery	0/22	0/4	NA

## Discussion

*C. posadasii/immitis* is endemic to the American Southwest and Central and South America [29]. About 60% of cases are asymptomatic [30]. Up to 30% of community-acquired pneumonia cases in endemic regions are attributed to *C. posadasii/immitis* [31]. In immunocompetent persons, 1% of infections progress to disseminated disease, mainly involving the skin, skeletal system, and CNS [30]. Risk factors for dissemination include immunosuppression, male sex, pregnancy, and African American or Filipino descent [29]. Azoles, especially fluconazole, remain the cornerstone of antifungal therapy. Other azoles and amphotericin B can be used in severe or refractory cases.

CIE is uncommon. Even with disseminated disease, cardiac involvement is rare and usually involves the pericardium [32] and myocardium. The incidence of myocarditis with disseminated coccidioidomycosis was 9% to 28% in autopsy studies [33]; none documented endocarditis. Furthermore, a review of 113 cases of *Coccidioides *fungemia by Keckich et al. [34] also reported no cases of established endocarditis.

CIE is difficult to diagnose for several reasons. It is rare, manifestations can be extremely heterogeneous, and although *Coccidioides *organisms grow well in most bacteriologic or mycologic media [35], fungemia is uncommon. A series of 150 cases of disseminated coccidioidomycosis documented only nine cases (0.06%) of fungemia [36]. Our cohort of 12 patients with CIE included only two with fungemia. In addition, *Coccidioides *can take an average of six days to grow in blood cultures [34]. This duration is beyond the five-day incubation for standard blood cultures. If *Coccidioides *fungemia is suspected, the laboratory should be alerted to extend the incubation of standard blood cultures. Alternatively, fungal blood cultures should be requested. It is imperative to alert lab personnel if *Coccidioides *is suspected and cultures should be handled in a biosafety level 3 laboratory.

Findings of echocardiography including TEE can be negative early in the course of CIE or after embolic episodes. If clinical suspicion remains high, serial echocardiography should be pursued. CF titers of 1:16 or higher can raise suspicion for disseminated coccidioidomycosis [35]. However, a specific titer did not correlate with a risk of, or predict, CIE in this small cohort. Our case patient and three others had a titer of 1:16 or greater, but the other cases of CIE had either lower titers [3,7,10] or a negative titer in a transplant recipient [11].

Our CIE cohort was predominantly young and male, which is consistent with the literature for disseminated coccidioidomycosis [37]. Most cases involved valvular rather than mural endocarditis; prosthetic valves were affected in two cases.

Blastomycosis is caused by the fungus *Blastomyces dermatitidis* or *gilchristii*. We acknowledge the recent emerging pathogen *Blastomyces *helicus, formerly known as *Emmonsia helicus* [38]. *Blastomyces *species are endemic in the Midwest, the Mississippi and Ohio River basins, the Canadian provinces bordering the Great Lakes, and a small area in New York. Blastomycosis most commonly manifests as an acute or chronic pneumonia with the potential for widespread dissemination. The most common sites of extrapulmonary infection are the skin, bones, and genitourinary system [39]. Risk factors for dissemination are immunosuppression, diabetes, and multilobar pulmonary disease [40]. Mild pulmonary and non-CNS extrapulmonary disease responds to itraconazole. More severe pulmonary and disseminated disease, and CNS blastomycosis, are best managed with initial liposomal amphotericin B followed by six to 12 months of itraconazole.

Similar to CIE, BIE occurs exclusively with disseminated disease. Fungemia is rare; the organism can take weeks to grow, and growth in standard blood culture is difficult even with prolonged incubation, the organism preferring specialized growth media [39]. In our BIE cohort, all patients had negative blood cultures. Diagnosis was made either by histopathologic analysis of endocardial tissue, or by echocardiography, and by tissue visualization of fungus from sites of dissemination. *Blastomyces *urine antigen testing performed in one patient was positive. *Blastomyces *serum serologic or urine antigen testing does have utility in disseminated blastomycosis [38].

Our cohort of BIE was mostly young men (median age, 24 years), with a median time to diagnosis of 10 months. Only one case was immunosuppressed. Eight of 11 cases of BIE were diagnosed before the routine use of echocardiography in the 1970s [41]. In contrast to CIE and HIE, only three cases of BIE had valvular endocarditis, all involving a native valve. The others had mural endocarditis, appearing as an extension of chest wall disease through the mediastinum to the heart. Therefore, in the setting of disseminated blastomycosis manifesting as intrathoracic, mediastinal, or chest wall abscess, echocardiography or additional imaging dedicated to cardiac structures is valuable in demonstrating cardiac involvement.

The direct contribution of BIE to mortality is unclear; in eight of 11 patients with disseminated disease, cardiac involvement was diagnosed post-mortem. Surviving patients with BIE were diagnosed in the 21st century. One received a combination of amphotericin B and itraconazole [26] and the other, an unnamed antifungal [25]. Neither required surgery.

*Histoplasma capsulatum* is the most common endemic mycosis in the United States [42]. It is predominantly found along the Mississippi and Ohio River valleys, although the geographic distribution of all endemic fungi in the US continues to expand [43]. Pulmonary infection can disseminate to the skin, bone marrow, CNS, gastrointestinal tract, adrenal glands, and heart. Most forms of histoplasmosis can be treated with itraconazole, but severe, disseminated disease warrants liposomal amphotericin B followed by itraconazole for at least one year.

HIE appears to be more common than CIE or BIE. The review of 61 cases of HIE by Boyanton et al. [1] had several interesting conclusions. HIE had extended diagnostic delays, and PVE cases were diagnosed at least 4.1 months earlier than native valve cases. Diagnosis of HIE was challenging; *Histoplasma *serum and urine antigen testing had a critical role in the early detection of disseminated disease. Fungal blood cultures were strongly recommended, and TEE was essential for diagnosis, especially in PVE. Surgery combined with effective and prolonged antifungal therapy remains the mainstay of treatment.

Our study has several limitations. First, our retrospective review encompasses cases of endemic fungal IE dating to the 1930s, with inconsistencies in disease description, management, and follow-up. Second, several cases predate currently available antifungal therapy and were diagnosed post-mortem. Hence, firm conclusions regarding outcomes and mortality cannot be extrapolated to the current era with the availability of effective antifungals, diagnostic testing, and surgical advances. However, a historical perspective is important in understanding a rare complication of endemic fungal infections in the United States, and it is reassuring that endocarditis remains an uncommon entity. Third, the standard-modified Duke Criteria could not be applied to all cases of cardiac involvement for the reasons cited above, because several cases preceded echocardiography and blood cultures were often negative. Despite these limitations, we have collated the most comprehensive review of endemic fungal IE to date and emphasize that endemic fungal IE remains a diagnostic and therapeutic challenge in the 21st century.

## Conclusions

IE remains an uncommon manifestation of endemic fungal infections in the United States. Clinicians must maintain a high index of suspicion in select cases of disseminated disease. Fungal blood cultures, echocardiography, and other imaging modalities such as cardiac CT and positron emission tomography (especially in the setting of prosthetic valve endocarditis) might facilitate early diagnosis. Prompt surgical intervention combined with maximal doses and prolonged use of antifungal therapy may improve outcomes in CIE and HIE. The utility of surgery in BIE is unknown.

## References

[1] Boyanton BL Jr, Boamah H, Lauter CB (2021). Native vs prosthetic valve Histoplasma capsulatum infective endocarditis: a case report and systemic literature review comparing patient presentation, treatment modalities, clinical outcomes, and diagnostic laboratory testing. Open Forum Infect Dis.

[2] Evans N, Ball HA (1929). Coccidioidal granuloma: analysis of fifty cases. J Am Med Assoc.

[3] Horng LM, Yaghoubian S, Ram A, Johnson R, Castro L, Kuo J, Deresinski S (2015). Endocarditis due to Coccidioides spp: the seventh case. Open Forum Infect Dis.

[4] Aronowitz P (2014). Cutaneous, pulmonary, and cardiac invasion in a young woman. Consultant.

[5] Epstein E (1938). Prognostic significance of cutaneous lesions in coccidioidal granuloma. Arch Derm Syphilol.

[6] Olavarria R, Fajardo LF (1971). Ophthalmic coccidioidomycosis. Case report and review. Arch Pathol.

[7] Reuss CS, Hall MC, Blair JE, Yeo T, Leslie KO (2004). Endocarditis caused by Coccidioides species. Mayo Clin Proc.

[8] La Via WV, Koulouri S, Ross LA, Quiros JA, Mason WH (2005). Right atrial mass in a child with disseminated coccidioidomycosis. Pediatr Infect Dis J.

[9] ME RK, LO DB, GE PH, ED JH, UT JP (1958). Fungal endocarditis: review of the literature and report of three cases. Ann Intern Med.

[10] TO TE, MC RW (1953). Coccidioidomycosis in infants. AMA Am J Dis Child.

[11] Nelson JK, Giraldeau G, Montoya JG, Deresinski S, Ho DY, Pham M (2016). Donor-derived Coccidioides immitis endocarditis and disseminated infection in the setting of solid organ transplantation. Open Forum Infect Dis.

[12] Abbott K, Cutler O (1936). Chronic coccidioidal meningitis: review of the literature and report of seven cases. Arch Pathol.

[13] Harding CV (1991). Blastomycosis and opportunistic infections in patients with acquired immunodeficiency syndrome. An autopsy study. Arch Pathol Lab Med.

[14] Pappas PG, Pottage JC, Powderly WG (1992). Blastomycosis in patients with the acquired immunodeficiency syndrome. Ann Intern Med.

[15] Kaplan W, Clifford MK (1964). Blastomycosis. I. A review of 198 collected cases in Veterans Administration hospitals. Am Rev Respir Dis.

[16] Larson RE, Bernatz PE, Geraci JE (1966). Results of surgical and nonoperative treatment for pulmonary North American blastomycosis. J Thorac Cardiovasc Surg.

[17] Wade HW, Bel GS (1916). A critical consideration of systemic blastomycosis: with notes on certain special features and report of five cases. Arch Intern Med.

[18] Hurley TD (1916). An unique lesion of the heart in systemic Blastomycosis. J Med Res.

[19] Bechtel RE, LeCount ER (1914). A case of systemic blastomycosis with necropsy. Arch Intern Med.

[20] Coupal J (1924). Report of six cases of blastomycosis. Int Clin.

[21] Medlar EM (1927). Pulmonary blastomycosis; its similarity to tuberculosis. Report of two cases. Am J Pathol.

[22] Baker RD, Brian EW (1937). Blastomycosis of the Heart: Report of Two Cases. Am J Pathol.

[23] Pond NE, Humphreys RJ (1952). Blastomycosis with cardiac involvement and peripheral embolization. Am Heart J.

[24] Bruce Light R, Kralt D, Embil JM (2008). Seasonal variations in the clinical presentation of pulmonary and extrapulmonary blastomycosis. Med Mycol.

[25] Richards LM, Adil A, Bajwa T, Khandheria BK (2018). Not just another Wisconsin case of blastomycosis. Eur Heart J Cardiovasc Imaging.

[26] Walker J, Biondi N, Pivato E, Sharma S, Phatak P, Das M (2023). Blastocyst or blastomycosis?: A rare presentation of disseminated blastomycosis with cardiac involvement in pregnancy. JACC Case Rep.

[27] Ashbee HR, Evans EG, Viviani MA (2008). Histoplasmosis in Europe: report on an epidemiological survey from the European Confederation of Medical Mycology Working Group. Med Mycol.

[28] Riddell J 4th, Kauffman CA, Smith JA (2014). Histoplasma capsulatum endocarditis: multicenter case series with review of current diagnostic techniques and treatment. Medicine (Baltimore).

[29] Kimes KE, Kasule SN, Blair JE (2020). Pulmonary Coccidioidomycosis. Semin Respir Crit Care Med.

[30] Galgiani JN, Ampel NM, Blair JE, Catanzaro A, Johnson RH, Stevens DA, Williams PL (2005). Coccidioidomycosis. Clin Infect Dis.

[31] Valdivia L, Nix D, Wright M (2006). Coccidioidomycosis as a common cause of community-acquired pneumonia. Emerg Infect Dis.

[32] Arsura EL, Bobba RK, Reddy CM (2005). Coccidioidal pericarditis: a case presentation and review of the literature. Int J Infect Dis.

[33] Albakri A (2019). Fungal cardiomyopathy: a review and pooled analysis of pathophysiology, diagnosis and clinical management. Res Rev Insights.

[34] Keckich DW, Blair JE, Vikram HR (2010). Coccidioides fungemia in six patients, with a review of the literature. Mycopathologia.

[35] Saubolle MA, McKellar PP, Sussland D (2007). Epidemiologic, clinical, and diagnostic aspects of coccidioidomycosis. J Clin Microbiol.

[36] Adam RD, Elliott SP, Taljanovic MS (2009). The spectrum and presentation of disseminated coccidioidomycosis. Am J Med.

[37] Brown J, Benedict K, Park BJ, Thompson GR 3rd (2013). Coccidioidomycosis: epidemiology. Clin Epidemiol.

[38] Schwartz IS, Wiederhold NP, Hanson KE, Patterson TF, Sigler L (2019). Blastomyces helicus, a new dimorphic fungus causing fatal pulmonary and systemic disease in humans and animals in Western Canada and the United States. Clin Infect Dis.

[39] Saccente M, Woods GL (2010). Clinical and laboratory update on blastomycosis. Clin Microbiol Rev.

[40] Villacorta Cari E, Leedy N, Ribes JA, Soria J, Myint T (2022). Risk factors of severe blastomycosis and comparison of diagnosis and outcomes between immunocompetent and immunocompromised patients. Mycoses.

[41] Maleki M, Esmaeilzadeh M (2012). The evolutionary development of echocardiography. Iran J Med Sci.

[42] Simms A, Kobayashi T, Endelman L, Sekar P (2020). Disseminated histoplasmosis presenting as bilateral lower extremity paresis. Int J Infect Dis.

[43] Mazi PB, Sahrmann JM, Olsen MA (2023). The geographic distribution of dimorphic mycoses in the United States for the modern era. Clin Infect Dis.

